# Inherited Retinal Dystrophies: Role of Oxidative Stress and Inflammation in Their Physiopathology and Therapeutic Implications

**DOI:** 10.3390/antiox11061086

**Published:** 2022-05-30

**Authors:** Isabel Pinilla, Victoria Maneu, Laura Campello, Laura Fernández-Sánchez, Natalia Martínez-Gil, Oksana Kutsyr, Xavier Sánchez-Sáez, Carla Sánchez-Castillo, Pedro Lax, Nicolás Cuenca

**Affiliations:** 1 Aragón Health Research Institute (IIS Aragón), 50009 Zaragoza, Spain; 2 Department of Ophthalmology, Lozano Blesa, University Hospital, 50009 Zaragoza, Spain; 3 Department of Surgery, University of Zaragoza, 50009 Zaragoza, Spain; 4 Department of Optics, Pharmacology and Anatomy, University of Alicante, 03690 Alicante, Spain; laura.fs@ua.es; 5 Alicante Institute for Health and Biomedical Research (ISABIAL), 03010 Alicante, Spain; pedro.lax@ua.es (P.L.); cuenca@ua.es (N.C.); 6 Department of Physiology, Genetics and Microbiology, University of Alicante, 03690 Alicante, Spain; laura.campello@nih.gov (L.C.); natalia.martinez.gil@ua.es (N.M.-G.); oksana.kutsyr@ua.es (O.K.); xsanchez@ua.es (X.S.-S.); carla.sanchez@ua.es (C.S.-C.)

**Keywords:** inherited retinal dystrophies, oxidative stress, inflammation, reactive oxygen species, clinical trial

## Abstract

Inherited retinal dystrophies (IRDs) are a large group of genetically and clinically heterogeneous diseases characterized by the progressive degeneration of the retina, ultimately leading to loss of visual function. Oxidative stress and inflammation play fundamental roles in the physiopathology of these diseases. Photoreceptor cell death induces an inflammatory state in the retina. The activation of several molecular pathways triggers different cellular responses to injury, including the activation of microglia to eliminate debris and recruit inflammatory cells from circulation. Therapeutical options for IRDs are currently limited, although a small number of patients have been successfully treated by gene therapy. Many other therapeutic strategies are being pursued to mitigate the deleterious effects of IRDs associated with oxidative metabolism and/or inflammation, including inhibiting reactive oxygen species’ accumulation and inflammatory responses, and blocking autophagy. Several compounds are being tested in clinical trials, generating great expectations for their implementation. The present review discusses the main death mechanisms that occur in IRDs and the latest therapies that are under investigation.

## 1. Introduction

Degenerative diseases of the central nervous system (CNS) are enigmatic and complex conditions that, despite their different etiologies, share common events, leading to neuronal death and irreversible loss of cognitive, visual, acoustic, or motor function. 

As the neural component of the eye, the retina is considered to be part of the CNS, and consists of several layers of perfectly organized and interconnected neurons and glial cells [1]. Light is captured by photoreceptors (rods and cones) to initiate the phototransduction cascade; visual information is then processed and sent to the brain, resulting in visual perception, allowing us to perceive the surrounding environment. The high metabolism and oxygen consumption intrinsic to photoreceptor cells leads to the production of free radicals, which must be in perfect balance with endogenous and exogenous antioxidant and anti-inflammatory mechanisms. This equilibrium is lost in the states of cell damage induced by retinal pathologies, leading to morphological and functional alterations that can be clinically observed using different methodologies, including optical coherence tomography and electrophysiological testing [2,3]. The central region of the human retina is the fovea, where the highest density of cones is concentrated. This region has a unique architecture [4], corresponds to the area of maximal visual acuity (VA), and is subject to great environmental stress.

Inherited retinal dystrophies (IRDs) are a group of progressive retinal degenerative disorders that drive the loss of visual function. They comprise many overlapping conditions, such as retinitis pigmentosa (RP), Leber congenital amaurosis (LCA), and Stargardt disease, among others. Together, IRDs have a prevalence of one case in every 3000 individuals [5], with hundreds of genes being linked to the diseases [6]. IRDs are typically classified based on the cell type that is predominantly affected and occur in both syndromic and non-syndromic forms. The most common form of rod dystrophy is RP, whereas the most frequent cone dystrophy is Stargardt disease. Although hundreds of genes have been identified as direct or contributing causes of retinal degeneration in IRDs, the predominant phenotype is an early loss of photoreceptor cells, which are unable to regenerate. During disease progression, all IRDs share basic common mechanisms with other CNS degenerative diseases, including oxidative stress, neuroinflammation and cell death. Microglial cells and, later, astrocytes and Müller cells, secrete multiple pro-inflammatory mediators including cytokines, chemokines, trophic factors, reactive oxygen species (ROS), nitric oxide (NO) and tumor necrosis factor alpha (TNF-𝛼), which promote chronic inflammation and are related to severe pathological side-effects (reviewed in [3]). Several drugs with antioxidant and/or anti-inflammatory properties have shown promising results both in vitro and in vivo for the treatment of IRDs [3,7]. Gene- and cell-based therapies are not yet commonly available, and no single drug has been proven to prevent or revert visual loss. Combined therapies of antioxidant, anti-inflammatory and antiapoptotic agents are a common pharmacological approach to slow down the degenerative processes, and to preserve visual function and quality of life. A promising design of new hybrid compounds with more than one mode of action is also under investigation. It is important to note that even a degenerated retina with a complete loss of vision can benefit from the administration of neuroprotective factors, which aid in the maintenance of other non-visual functions, such as the control of circadian rhythms and pupil contraction [8]. 

In this review, we will focus on the effects of oxidative stress and inflammation in the pathogenesis of IRDs and neuroprotective approaches that slow vision loss caused by oxidative damage and pro-inflammatory processes.

## 2. Definition of Oxidative Stress and Inflammation

### 2.1. Oxidative Stress

Oxidative stress is defined as an imbalance in the formation and removal of ROS, which occurs when the production of free radicals, atoms or molecules that have an unpaired electron cannot be counteracted by antioxidant responses [9]. Oxygen- and nitrogen-containing species are susceptible to these energetic changes, which can occur as a result of cellular metabolism, generating ROS and reactive nitrogen species (RNS) [10]. Reactive oxygen and nitrogen species (RONS) can react with other stable molecules (e.g., proteins, lipids), promoting their loss of function and destabilizing cellular homeostasis [10]. Accordingly, oxidative and nitroxidative stress involves not only redox-mediated reactions, but also essential metabolic pathways [11,12]. In this review, the term oxidative stress will be used to describe both oxidative and nitroxidative stress. 

Different subcellular sources of RONS have been established, including mitochondria, endoplasmic reticulum (ER), microsomes and peroxisomes, and they can be generated by cytosolic and plasma membrane enzymes. Depending on the source, it is possible to differentiate the main reactive species that are produced [13]. Mitochondria are one of the major ROS producers, generating great quantities of not only superoxide anion radicals (O_2_•−) as a by-product of the electron transport chain, but also hydrogen peroxide (H_2_O_2_) [14]. Beyond the mitochondria, the transmembrane NADPH oxidases (NOXs) also generate O_2_•− and/or H_2_O_2_, depending on their location and regulation [13]. The ER and peroxisomes contain various types of oxidases that are also able to generate O_2_•− and H_2_O_2_ [15,16]. H_2_O_2_ is recognized as the major ROS in redox signaling pathways [13], and is produced by the dismutation of O_2_•− (which is short-lived) to H_2_O_2_ (which is more stable) by cytosolic, mitochondrial, and nuclear superoxide dismutase (SOD) enzymes [17]. Despite the diverse cellular sources of H_2_O_2_ and its ability to modulate different redox cellular responses, it is less reactive than the hydroxyl radical (•OH), which is the most reactive ROS. •OH is formed by the interaction between H_2_O_2_ and metal ions (Fe^2+^ or Cu^2+^) through Fenton reactions at or close to its site of formation. In a similar reaction to that needed for oxygen to generate ROS, reactive nitrogen intermediates are successive 1-electron reduction products of NO (NO•) [18]. NO, which is an important signaling molecule, is generated by three isoforms of NO synthase (NOS) that can be found in different subcellular compartments, including the plasma membrane, Golgi apparatus, mitochondria, cytosol, or peroxisomes [19,20,21]. Each NOS isoform is predominantly found in a specific tissue or context: NOS1 is also named neuronal NOS (nNOS) because it is constitutively expressed in neural tissue; NOS3 is also named endothelial NOS (eNOS), as it is mostly found in endothelial cells; NOS2 is also named inducible NOS (iNOS), as it is upregulated by pro-inflammatory factors [22]. As NO plays a key role in vascular homeostasis and neural activity, it cannot be considered a toxic RNS; however, its reaction with O_2_•− generates peroxynitrite (ONOO−), which is a powerful oxidant [23]. Accordingly, when NO is inefficiently removed and superoxide anion production is elevated, the consequent generation of ONOO−can damage many biological molecules, such as proteins, lipids, and nucleic acids [23].

### 2.2. Inflammation

Inflammation is a protective response against injury, infection, or other harmful stimuli. An inflammatory state is a common scenario in IRDs [3]. The most important retinal immune cells are the microglial cells, which are responsible for maintaining retinal homeostasis and mediating the inflammatory response in the retina. Additionally, macroglial cells, which include Müller cells and astrocytes, also participate to preserve retinal health [24]. A balance is maintained between pro-inflammatory and anti-inflammatory stimuli under healthy conditions; however, in degenerating tissue, a plethora of pro-inflammatory molecules are secreted, first by microglia and later by Müller cells and astrocytes, changing their primary defensive response into a chronic inflammatory condition that aggravates the already-poor retinal health [3]. 

The various harmful stimuli are detected by retinal immune cells through engagement with pattern recognition receptors, including pathogen-associated molecular patterns and damage-associated molecular patterns, which respond by upregulating myriad inflammatory genes [25]. Four classes of pathogen recognition receptor families have been described to date: Toll-like receptors, C-type lectin receptors, retinoic acid-inducible gene (RIG)-I-like receptors, and NOD-like receptors [26]. The activation of these receptors triggers a change in retinal microglia morphology, from a normal, highly ramified morphology to an amoeboid morphology, and also modifies their secretory phenotype to pro-inflammatory or anti-inflammatory [27]. Similar to other degenerative CNS diseases, the major contributors to IRD-related inflammation and cell death are TNF-𝛼 and interleukin (IL)-1. TNF-𝛼 is a key factor secreted in the retina by activated microglia and macrophages [25] and can trigger different cell-death mechanisms, including apoptosis, pyroptosis, necroptosis, and parthanatos [25,28]. 

The most common pathways that are activated in IRDs are the nuclear factor kappa-beta (NF-κB), Janus kinase/signal transducer and activator of transcription (JAK/STAT), and mitogen-activated protein kinase (MAPK) signaling pathways, ultimately leading to the release of pro-inflammatory molecules [28]. The NF-κB signaling pathway is stimulated through different receptor subsets, including pro-inflammatory cytokines, growth factors, lipopolysaccharide (LPS), and antigen receptors, which activate the IkB–kinase complex. This activation, in turn, triggers the phosphorylation, ubiquitination and degradation of IkB proteins, releasing the p50 and p65 subunits, which translocate to the nucleus and activate the transcription of pro-inflammatory genes [29,30,31]. Alterations in NF-κB expression have been reported in different retinal degeneration conditions [28,29]. Activation of the JAK/STAT signaling pathway, through STAT phosphorylation by dimerized JAK, has also been reported to be involved in many neurodegenerative diseases, including RP. Activated STAT is translocated to the nucleus, where it regulates the expression of numerous genes. IL-6 is one of the best-known activators of the JAK1/STAT3 pathway in macrophages [32,33,34,35]. Finally, the MAPK family (p38-MAPK, c-Jun N-terminal kinase -JNK, and extracellular signal-regulated kinases-ERK1/2) also plays an important role in retina cell survival and apoptosis, and a variety of cytokines can activate this pathway, leading to the release of neuroinflammatory cytokines [36,37,38]. 

### 2.3. Effects of Oxidative Stress and Inflammation in Inherited Retinal Dystrophies 

The retina is one of the most metabolically active tissues in the body and is exposed to high levels of light and oxygenation. Accordingly, the RONS flux needs to be a perfectly regulated process under steady-state conditions. Oxidative stress occurs when the balance between RONS formation and its removal is disrupted, which activates inflammation and cell-death pathways, and retinal degeneration. It has been demonstrated that oxidative stress plays a central role not only in the most common eye diseases, such as glaucoma [39,40], age-related macular degeneration (AMD) [41,42], and diabetic retinopathy (DR) [43], but also in IRDs [44].

IRDs are characterized by the progressive degeneration of the retina, the retinal pigment epithelium (RPE), and the choriocapillaris, the vascular source of nutrients and oxygen. A total of 280 genes have been directly related to retinal dystrophies to date, with photoreceptors and RPE cells serving as the main carriers of the mutations [6]. While there are common mechanisms through which oxidative stress and inflammation trigger retinal neurodegeneration in these retinopathies, affecting cellular metabolism, cell signaling and homeostasis, it is still unknown whether oxidative stress is the main cause of the pathology, or whether it is a secondary contributor to its progression [45].

Retinal degeneration in IRDs involves inflammation, oxidative stress and cell death. In this situation, a non-ocular stimulus that stimulates inflammation can exacerbate photoreceptor cell death, which worsens the degenerative process. This was shown for P23H rats (an RP model with a mutation in the rhodopsin gene) administered systemically with LPS, which increased the expression levels of several inflammation-related genes such as TNF-𝛼, IL-1𝛼, IL-1𝛽 and caspases-3 and -8 [46]. Other factors, including a high-fat diet, can worsen the disease progression in rd10 mice (a model of autosomal RP whose mutation in phosphodiesterase 6 (PDE6b) is the same as that found in patients with RP) through the increased expression of inflammatory mediators such as glycogen synthase kinase-3β (GSK-3β), NF-κB and IL-6, phospho-STAT3/STAT3 and IL-1α [35]. 

#### 2.3.1. Purinergic Signaling Activation

Purinergic signaling, particularly through the P2X7 receptor (P2X7R), plays a fundamental role in the evolution of the retinal degenerative process [47]. Most purinergic receptor subtypes are expressed in the mammalian retina. P2X7R can be found in the RPE, astrocytes, microglia, Müller cells, and in pericyte-containing retinal microvessels [48,49,50,51,52,53]. The expression of P2X7R has also been reported in the synaptic terminals of cones and rods, the inner plexiform layer, and retinal ganglion cells [51,52,54]. P2X7R is an ATP-gated ion channel that, when activated by ATP secreted from neurons or glial cells, leads to the activation of microglial cells, which release pro-inflammatory cytokines that help to extend the gliosis (cellular response to injury) to surrounding tissue [3,47,55,56]. When overactivated, P2X7R mediates the formation of wide plasma membrane pores that contribute to calcium overload and cytotoxicity. The P2X7 pore is also associated with the activation of the inflammasome and with the inflammasome-dependent cell-death pathway [57], leading to the release of pro-inflammatory cytokines and ROS by macrophages and microglia [58]. In the P23H rat model of RP, inflammasome activation was found to be a major cause of cone photoreceptor death, mediated by P2X7R [59]. In the rd1 mouse model of RP, the inhibition of P2X receptor signaling delays photoreceptor degeneration [60].

#### 2.3.2. Cell Death 

##### Apoptosis

For many years, apoptosis was considered to be the only mechanism of programmed cell death, whereas necrosis was defined as an unregulated process. However, new programmed cell-death mechanisms have been described for IRDs, with oxidative stress playing a major role in their induction [28,61]. The intracellular machinery responsible for apoptosis involves a family of caspase proteases, which are synthesized in the cell as inactive precursors (or procaspases) and are activated by either extracellular or intracellular stimuli. As an intrinsic apoptosis pathway, elevated intracellular oxidative stress up-regulates pro-apoptotic markers such as Bax family members, which induce the expression and activation of caspases- 3, 6, 7 and 9. By contrast, the extrinsic pathway is activated by members of the TNF receptor family that are also modulated by oxidative stress and inflammation mediators, inducing the expression and activation of caspases- 3, 6, 7, and 8 [28]. Both extrinsic and intrinsic activation lead to DNA fragmentation and cytoplasm degradation, ultimately promoting cell death [61].

##### Necroptosis

Although traditionally classified as a type of uncontrolled cell death process, different types of regulated necrosis that trigger cell death have been reported in retinal dystrophies, including necroptosis, pyroptosis, parthanatos and ferroptosis [61]. Necroptosis is induced by the TNF-α pathway and is mediated by the interaction between protein kinase-1 receptor (RIPK1) and RIPK3. When caspases are inactivated, the formation of the RIPK1/RIPK3 necroptosis complex activates the mixed-lineage kinase domain-like (MLKL) protein, which promotes plasma membrane permeability. In the context of RP, RIPK-mediated programmed necrosis not only plays a critical role in inducing cell death in RPE and cone photoreceptors, but also promotes retinal inflammation during retinal degeneration [62].

##### Parthanatos

Several authors suggest that parthanatos contributes to retinal neurodegeneration in different RP models [63]. Photoreceptor cell death mediated by parthanatos begins with an oxidative stress or inflammatory stimulus that triggers the activation of poly-ADP-ribose polymerase (PARP) and Ca^2+^-dependent cysteine proteinases (calpains). Subsequently, the release of apoptosis-inducing factor (AIF) from mitochondria to the cytoplasm ultimately results in DNA fragmentation [64,65]. Under physiological conditions, cells have different mechanisms to regulate parthanatos. However, in a scenario of retinal neurodegeneration, the upregulation of PARP activity and cGMP signaling, together with cytosolic Ca^2+^ overload, culminates in cell death [66].

##### Pyroptosis

Whereas necroptosis and parthanatos are caspase-independent mechanisms of cell death, pyroptosis occurs upon the activation of pro-inflammatory caspases [67]. Pyroptosis promotes cellular lysis by disturbing the electrochemical gradient and the osmotic potential of cellular membranes. Following inflammasome activation, caspase-1 cleaves and activates the gasdermin (GSDM) family of pore-forming proteins, of which gasdermin D (GSDMD) is the main pore-forming executioner in the plasma membrane [67]. This inflammatory-induced cell death mechanism has been described in different neurodegenerative diseases, such as Alzheimer’s disease [68]. Moreover, there is evidence of its possible involvement in photoreceptors and RPE cell death in experimental IRDs models [69]. However, the cellular mechanisms by which it occurs are unknown and further investigation is needed. 

##### Ferroptosis

Ferroptosis is another cell-death mechanism associated with CNS disease and has recently been linked to retinal degenerative processes in experimental RP models [70,71]. As the name suggests, this programmed cell death pathway is dependent on iron, and is induced by the oxidation of polyunsaturated fatty acids (PUFAs) that are present in lipid bilayer membranes. Iron has been described to act as a catalyst in the Fenton reaction (where H_2_O_2_ is converted to ·OH radicals), triggering a free-radical chain reaction and resulting in PUFA oxidation or degradation [72]. The iron-dependent oxidation of PUFAs causes mitochondrial alterations and cell membrane damage, which promotes cell death. In this line, it has been proposed that the dysregulation of heme or iron homeostasis due to variations in a gene encoding a heme-transporter protein is a cause of RP [73]. Accordingly, defects in iron metabolism not only could promote ferroptosis, but can also induce retinal degeneration. 

#### 2.3.3. Autophagy/Mitophagy and Mitochondrial Effects 

Autophagy is an essential mechanism in the retina to ensure correct retinal development function and tissue homeostasis by removing damaged cell components [74,75,76]. In the context of IRDs, autophagy has mostly been studied in photoreceptors and RPE cells, likely due to its implications in the visual cycle [77]. Autophagic activity is involved in the degradation of the visual pigments and in adaptation to circadian rhythms and is essential to the maintenance of protein concentrations that are implicated in the visual cycle [77]. Moreover, in RPE cells, autophagy degrades lipofuscin (age pigment) aggregates and vesicles that contain the photoreceptor outer segment membranes after shedding [78].

Beyond its role in retinal cell homeostasis, autophagy is also activated under oxidative stress conditions. It is a regulated catabolic process where damaged organelles such as mitochondria (termed mitophagy), misfolded proteins or waste products of cellular metabolism are internalized in autophagosomes, to be later degraded through fusion with lysosomes. The activation of autophagy requires the inhibition of the nutrient sensor molecular target of rapamycin (mTOR) and the activation of the energy sensor 5-AMP-activated protein kinase (AMPK). The formation and elongation of the autophagosome requires the participation of different proteins, including ATG proteins, BECLIN1 and LC3. The fusion of autophagosomes containing dysfunctional cargo with a lysosome allows for the degradation or recycling of the content [74]. 

The role of autophagy in the retina and its implications for retinal degeneration has been studied in several different experimental models [79,80,81]. Autophagy appears to be blocked in rd10 mice, suggesting that its activation could play a neuroprotective role [78]. By contrast, studies in P23H rats revealed that the upregulation of autophagy could be related to an increase in photoreceptor death and proteasome blockage [82,83]. Therefore, autophagy likely plays a dual role: maintaining retinal homeostasis in healthy conditions and participating in retinal degeneration processes in disease states.

#### 2.3.4. Lipid Peroxidation

Lipid peroxidation is an important oxidative-stress-related event associated with retinal damage and pathogenesis [44]. It is a metabolic pathway that can be over-activated not only under cellular stress, but also under chronic light exposure, inducing photooxidative damage [84,85,86,87]. Mechanistically, RONS accumulation promotes the oxidation of unsaturated lipids, especially PUFAs present in the lipid bilayer membranes of the cells, resulting in their oxidative degradation to a variety of products. 

Photoreceptors contain large quantities of PUFAs, especially in their outer segment membranes. An increase in lipid peroxidation products in cones was reported in a pig model of RP [61]. Moreover, because RPE cells are responsible for photoreceptor outer segment shedding, the accumulation of lipid protein aggregates (i.e., lipofuscin) and lipid peroxidation products (i.e., acrolein and 4-hydroxynonnenal (4-NHE)) has been suggested to contribute to retinal degeneration in some IRDs, such as Best or Stargardt diseases [88,89,90]. There is a close relationship between the increase in lipid peroxidation processes and the activation of different cell death pathways in retinal dystrophies. Lipid peroxidation not only induces apoptosis and autophagy [91], but it has recently been demonstrated that lipid peroxidation products react with iron molecules in RP, which promotes photoreceptor cell death by ferroptosis [70,71]. 

#### 2.3.5. Nucleic Acid Damage

Nuclear DNA is less vulnerable than other DNA structures to increases in RONS; however, it can be directly damaged by a chemical attack from purine and pyrimidine bases and deoxyribose sugars [23]. Moreover, RONS accelerates the shortening of chromosome telomeres [44]. By contrast, the close proximity of mitochondrial DNA to ROS generation in the inner mitochondrial membrane undoubtedly increases its vulnerability to oxidation and damage. In this line, the upregulation of DNA polymerase gamma and 8-oxoguanine-DNA-glycosylase, which are selectively localized in the mitochondria of the photoreceptor synaptic terminals and exert a DNA-repair function, has been proposed as a pharmacologic strategy to promote photoreceptors’ rescue in degenerative retinal diseases [92].

For decades, it has been proposed that RNA oxidation may occur during the early stages of degeneration in neurodegenerative diseases such as Alzheimer’s and Parkinson’s disease [93,94]. As RNA is generally single-stranded, it is considered more unstable than DNA. In addition, RNA comprises about 80–90% of the total nucleic acids in cells and is localized in the cytoplasm, adjacent to mitochondria, where more free radicals are generated. Thus, RNA is considered more vulnerable to oxidative damage than DNA or proteins. Hydroxyl radicals are the major RNA oxidants and 8-hydroxyguanosine (8-OHG) is the most prevalent oxidized nucleobase (C-8 position of deoxyguanosine) in RNA by •OH [93]. RNA oxidative damage affects the translational process during protein synthesis, causing truncated, misfolded and/or aggregated proteins [93]. Furthermore, alterations in the expression profile of long non-coding RNAs and short non-coding RNAs (such as some micro-RNAs) have been directly related to the increase in oxidative stress, not only in the neural retina, but also in the RPE [58].

#### 2.3.6. Protein Damage and Endoplasmic Reticulum Stress

The ER acts as a quality-control organelle that retains and degrades misfolded proteins. Increases in intracellular RONS induce protein metabolism and structure disorders through the oxidation of and reduction in cysteine residues, the formation of protein–protein cross-linkages, or by post-translational modifications [94]. These changes can lead to secondary effects, including protein fragmentation, aggregation, and unfolding [95] which result not only in the loss of protein function but also in a decline in cellular antioxidant capacity and the degradation of oxidized or misfolded proteins by the proteasome or autophagy processes. In the retina, under uncontrollable oxidative and nitro-oxidative stress conditions, the ER can activate cell-death pathways, such as apoptosis [96], as a protective response, promoting the neurodegenerative process. Moreover, ER stress has been suggested to be a primary cause of photoreceptor death associated with rhodopsin mutations [97].

## 3. Therapeutic Strategies to Reduce Oxidative Stress and Inflammation: Antioxidants and Anti-Inflammatory Agents and Other Strategies

At present, no pharmacological agent has been proven to be effective in preventing or restoring vision loss in IRDs. Accordingly, new therapies, including gene- and cell-based therapies, are urgently needed. Gene therapy could treat the disease or block its progression, depending on the moment of treatment and the gene mutation. Voretigene neparvovec is indicated for the treatment of patients with biallelic *RPE65* mutations associated in the interim; other strategies are being investigated to protect the retina and slow disease progression [98]. Neuroprotection aims to stop photoreceptor loss during IRD. Treatments that do not specifically target the molecular mechanisms of the disease could be used not only in IRDs, but also in other acquired retinal degeneration diseases, such as AMD or DR, the main causes of visual loss. Other ancillary treatments could be used to treat IRD complications such as macular edema or cataracts. In this section, we will specifically focus on neuroprotective interventions that slow the vision loss caused by oxidative stress and pro-inflammatory processes associated with retinal disease.

Neuroprotective strategies include a broad array of approaches that promote neuron survival by preserving their structure and function. A multitude of neuroprotective agents, including antiapoptotic, antioxidant and anti-inflammatory drugs, and rehabilitative methods such as exercise and electrical stimulation, have proven effective in animal models of retinal degeneration and in patients with reduced vision [3,99,100]. While none of these agents have been approved by the US Food and Drug Administration as drugs with neuroprotective effects, numerous compounds have been tested in research and in (pre)clinical studies [101,102]. While oxidative mechanisms underlie the pathophysiology of many IRDs, the sources and impact of RONS can depend on the pathogenic mutation, which will determine the effect of different drugs in preventing cone death and prolonging rod survival [44]. Mutations that cause the misfolding of transmembrane proteins involved in photoreception or phototransduction, such as rhodopsin, arrestin, PDE6 and TULP1, are associated with increased ER stress. The ER responds to the burden of misfolded proteins by activating the unfolded protein response (UPR), a complex intracellular mechanism that regulates the expression of multiple genes to maintain ER homeostasis and prevent further cell damage [103]. Genetic and pharmacologic interventions to modulate ER stress and UPR-associated transcriptional programs are promising technologies to treat retinal degeneration [104]. For example, the overexpression of chaperones such as BiP/Grp78 [105] and the downregulation of the UPR transcription factor ATF4 [106] have beneficial effects in cell culture or animal models of IRDs. Importantly, RPE cells show altered microRNA (miRNA) expression profiles under oxidative stress, and many of the dysregulated miRNAs are modulators of the expression of the causative genes of IRDs, such as *KLHL7*, *RDH11*, *CERKL*, *AIPL1* and *USH1G* [107]. 

While the existence and negative effects of oxidative stress in the etiopathogenesis of retinal dystrophies is clear, the translation from animal research into the clinic has many challenges. IRDs are rare diseases affecting a small fraction of the population, and there are countless genetic and phenotypic variations. In addition, after rod cell death, cones are chronically exposed to oxidative stress, which makes the design of long-term treatments necessary. Dietary supplementation and treatments with antioxidant and/or anti-inflammatory compounds have been shown to have beneficial effects in retinal dystrophies [3,44]. Below, we discuss potential candidates with promising neuroprotective properties in retinal disease. Table 1 lists most of the proven therapeutic interventions for IRDs and other retinal damage models, including AMD, DR and ganglion cell damage. Table 2 describes different clinical trials (CTs), which are ongoing and completed.

### 3.1. Antioxidants and Anti-Inflammatory Agents

#### 3.1.1. N-Acetylcysteine 

N-acetylcysteine (NAC) is clinically used as a mucolytic agent and as an antidote to acetaminophen toxicity. It is a sulfhydryl-containing liposoluble molecule that can penetrate cell membranes and has oral bioavailability. NAC is derived from the thiol-containing amino acid L-cysteine. It is a precursor of glutathione, and thus enhances glutathione S-transferase activity. NAC has a direct antioxidant effect via its reactive sulfhydryl agent; it can neutralize ROS by itself and can also break thiolated proteins, releasing free thiols and reduced proteins with antioxidant effects, such as mercaptoalbumin. NAC can stabilize protein structures by crosslinking cysteine disulfide molecules [445,446]. Its systemic administration results a significant intraocular concentration, although there is variability in its absorption. NAC has been studied in different eye conditions, including IRD, AMD, DR, glaucoma and Sjogren’s disease. A phase 1 randomized trial demonstrated that NAC is safe and well-tolerated and may improve sub-optimally functioning macular cones in advanced forms of RP [122]. 

Several CTs have been conducted to assess the effect of NAC in RP. The FIGHT-RP1 study (NCT03063021) was a phase-1 dose-escalation trial to test the effects of oral NAC in patients with RP. The primary outcome was safety, and secondary outcomes were assessed visual function, including best-corrected visual acuity (BCVA) and macular sensitivity using microperimetry. Patients were treated with escalating doses of NAC (600, 1200 or 1800 mg twice daily for 3 months); the highest NAC dose reduced the risk of macular sensitivity loss [121]. In the aforementioned CT, patients receiving NAC were divided into two arms depending on the carbonyl content GSH/GSSG level in aqueous humor. Another goal of FIGHT-RP1 is to study gene expressions that can interfere with NAC efficacy. In patients with idiopathic pulmonary fibrosis, polymorphisms in TOLLIP can influence the outcomes of NAC treatment. In the CT, DNA samples will be collected to identify the modifier genes that can impact cone survival. 

The FIGHT-RP1 extension study (NCT04864496), will use NAC 1800 mg twice daily to check safety and tolerability over 2 years of follow-up in patients with moderately advanced RP. Secondary outcomes will be measured, such as BCVA, central sensitivity using microperimetry, changes in the ellipsoid zone, changes in the aqueous GSH/GSSG ratio, and NAC and carbonyl levels. The estimated study completion date is the end of 2025. NAC appears to be well-tolerated, with minimal side-effects. While the most effective dosage is still unclear, its efficacy has been proven in multiple diseases that manifest with oxidative stress. Although similar to other antioxidants, caution needs to be exercised in lung cancer as a result of p53 inhibition [446]. 

#### 3.1.2. NPI-001 (N-Acetylcysteine Amide)

GMP-grade N-acetylcysteine amide (NPI-001, NACA) is the amide form of NAC manufactured by Nacuity Pharmaceuticals (Fort Worth, TX, USA). Its structure is more lipophilic, which allows for it to permeate cell membranes more than NAC while preserving its antioxidant properties [447,448]. It has demonstrated the ability to cross blood–brain and blood–retinal barriers with therapeutic potential in neurodegenerative diseases [447]. The compound is safe and effective. The SLO-RP study (NCT04355689) is a phase 1, 2 randomized CT using NPI-001 for RP associated with Usher syndrome; in phase 1, NPI-001 250 mg will be compared with placebo. 

#### 3.1.3. Thioredoxin

The thioredoxin (TRX) family of oxidoreductases contains the active center cysteine–glycine–proline–cysteine and oxidized cysteine groups. TRXs can protect against oxidative stress and also have anti-inflammatory properties [449,450,451]. Their antioxidant properties are based on the reduction in other proteins by cysteine thiol disulfide exchange [126]. Rod-derived cone viability factor (RdCVF) is a truncated (alternatively spliced) product of *NXNL1* (nucleoredoxin-like 1), which is secreted by rods and protects cones from degeneration through its actions as a trophic factor. The loss of rods in IRDs implies the lack of a protective effect of RdCVF, and the exogenous administration of this molecule has therapeutic value in preventing photoreceptor degeneration [452,453]. The dominant protein encoded by *NXNL1* is RdCVFL (TRX-like protein RdCVF) an enzymatically active TRX [454,455]. The loss of RdCFVL expression leads to increased oxidative damage, as reported in mice lacking *Nxnl1* [456]. In rd mice, TRX can protect against photoreceptor death, activates the GSH system, and partially reduces retinal gliosis [126].

#### 3.1.4. Saffron 

Saffron is the dried stigma (part of the pistil) of *Crocus sativus* L., and has a neuroprotective effect against oxidative damage. Its major constituents, crocin and crocetin, derive from carotenoids, and exert potent antioxidative actions due to multiple C-C bonds. Saffron has a complex mechanism of action beyond its antioxidant activity, as it also stimulates the mechanisms of tissue resilience [457]. Crocetin prevents retinal degeneration secondary to oxidative and ER stress by inhibiting caspase 3 and 9 activity in cell lines and in a mouse model of light retinal damage [458]. Crocin protects cultured photoreceptors from light damage [459]. Crocetin also increases oxygen diffusivity through tissues [460]. Metabolites of this flavonoid also directly bind to DNA, partially changing it to a beta-DNA conformation and protecting the cell from damage [461]. Saffron is also able to modulate gene expression involved in the retina response to light damage, including *endothelin 2* and *FGF* [129]. 

Saffron supplementation has been tested in the STARSAF02 trial for Stargardt disease (NCT01278277) [427]. In a short-term administration (6 months) study, 31 patients with *ABCA4* mutations were treated either with saffron or with placebo. The substance was well-tolerated but no improvement was observed in visual function. The study suggested that saffron may prevent the deterioration of macular flicker electroretinogram (ERG) amplitude, but a long-term study is needed to test its benefits [427]. The authors followed some patients for 36 months outside the period of the CT, finding that long-term supplementation could stabilize visual function, but these results need to be validated. Saffron has been also tested in a CT (NCT00951288) of early AMD, where it was found to stimulate an increase in ERG values against placebo and improve flicker sensitivity [134].

#### 3.1.5. Minocycline

Minocycline is a broad-spectrum, second-generation, semi-synthetic tetracycline with free-radical scavenging properties, and anti-inflammatory, antiapoptotic and neuroprotective properties [462]. While its mechanism of action is not fully understood, it appears to regulate several main enzymes and systems; for example, it downregulates NF-κB [463] and inhibits many enzymes, including iNOS, MMPs, phospholipase A2, caspases, and other proteins involved in apoptosis, including p38-MAPK and poly [ADP] ribose polymerase1 [462]. Minocycline has demonstrated neuroprotective effects in preclinical studies of neurodegenerative disorders, such as Alzheimer’s disease, Parkinson’s disease, Huntington’s diseases, amyotrophic lateral sclerosis and multiple sclerosis [462]. As an inhibitor of microglial activation, it has been shown to protect retinal ganglion cells (RGC) in mouse models of glaucoma [163,164]. In CTs with patients with neurodegenerative disorders, its effects have been modest at best [464,465]. At present, it is being tested in phase 2 CTs for treatment of AMD (NCT02564978) and RP (NCT04068207). In a CT testing its potential efficacy for the treatment of macular edema associated with RP, it failed to induce significant changes in mean visual acuity; however, a small but progressive decrease in mean central macular thickness was observed [428]. Minocycline can easily pass through the blood–brain barrier and is generally safe and well-tolerated. Adverse reactions include gastrointestinal distress and photosensitivity, hepatotoxicity, and an exacerbation of pre-existing renal failure; therefore, the monitoring of patients is recommended after 6 months of treatment [466]. 

#### 3.1.6. Melatonin 

Melatonin (N-acetyl-5-methoxytryptamine) is a hormone derived from the essential amino acid tryptophan and is synthesized by numerous organs including the pineal gland and the retina [467]. Melatonin is also found in edible plants and foods, including milk, fruits, vegetables, and fish, and in the medicinal herbs of chamomile (*Matricaria chamomilla* L.) and St John’s wort (*Hypericum perforatum* L.), among others [468,469]. Melatonin plays a major role in the regulation of circadian rhythms, in addition to modulating various physiological functions, such as cardiovascular and immune system regulation and retinal function [470]. Melatonin exhibits a high antioxidant capacity, directly acting as a free-radical scavenger, and indirectly enhances antioxidant enzymes, such as CAT, SOD, glutathione reductase, and GPX. Melatonin also suppresses the activity of pro-oxidant enzymes such as NOS and COX-2, and acts as a cell-survival agent by modulating autophagy [471].

Melatonin supplementation (in drinking water) in P23HxLE rats, a model that resembles the clinical features of heterozygous patients with RP, improved visual acuity, contrast sensitivity and retinal electrical activity [168]. It also improved circadian synchronization and increased the levels of CAT and SOD, which further reduced oxidative stress. Therefore, melatonin promotes cell survival, preserves retinal cells, and delays the progression of RP. 

The intravitreal administration of melatonin is also effective in ameliorating photoreceptor degeneration induced by the DNA-alkylating agent N-methyl-N-nitrosourea (MNU). Melatonin mediated the protective effects on visual function by modulating apoptotic cascades and alleviating oxidative stress [172]. Specifically, the retinas from mice treated with melatonin after MNU-induced damage showed lower levels of lipid peroxidation (reduced malondialdehyde, MDA) and DNA oxidation (decreased nuclear 8-OHdG) and higher levels of SOD and manganese SOD (MnSOD) than untreated counterparts [172].

#### 3.1.7. Curcumin 

Curcumin is a yellow polyphenolic pigment derived from the rhizome of turmeric (*Curcuma longa* L.) with well-known anti-inflammatory and antioxidant properties that are secondary to the inhibition of TNF-dependent NF-κB and pathways that produce ROS. Other actions include the downregulation of COX-2 and the inhibition of the pro-inflammatory enzyme 5-lipoxygenase, thus reducing the biosynthesis of inflammation products such as leukotrienes and lipid mediators. Curcumin also lowers the production of inflammatory cytokines including TNF, IL-1, IL-6. IL-8, iNOS and interferon-γ, and downregulates the expression of genes involved in apoptosis and cellular proliferation and inflammation [472]. Curcumin has pleotropic effects in different diseases, including gastrointestinal, cardiovascular, rheumatological and neurodegenerative diseases, including ocular pathologies (for a review, see [473]).

The effects of curcumin have been studied in cell lines and animal models of different retinal diseases, including IRDs. However, its clinical use is limited by its poor solubility and oral bioavailability, and different delivery mechanisms (liposomes and nanoparticles) are currently being studied [474,475]. Curcumin was protected against retinal degeneration in rat models of light-induced retinal degeneration by inhibiting NF-κB signaling [193]. In different retina cell lines, curcumin protected against H_2_O_2_ damage, altered the expression of H_2_O_2_-modulated miRNAs, increased the levels of antioxidant genes and reduced the expression of angiotensin II type 1 receptor, VEGF and NF-κB [476]. Curcumin also exhibits anti-protein aggregate activities in vitro, and improves retinal structure and function in P23H rats [191].

#### 3.1.8. Carotenoids 

The retina stores antioxidant products as macular pigments, including lutein, zeaxanthin and meso-zeaxanthin (converted from lutein in the retina) [477]. Zeaxanthin is present in the central region, whereas lutein concentration increases with radial distance from the fovea and meso-zeaxanthin levels decrease [477,478]. Lutein/zeaxanthin are related to static indicators of visual function [479], absorb blue light, scavenge ROS using their double bounds, protect membrane phospholipids against UV-induced peroxidation, induce phase-II antioxidant enzymes, and reduce lipofuscin formation [480]. Lutein suppresses NF-κB activation and the expression of iNOS and COX-2 [480]. The role of lutein or zeaxanthin in AMD has been studied in some depth. Genes controlling SR-B1 (a lutein-binding protein in the retina) and high-density lipoprotein levels predisposed to AMD, supporting the involvement of both lutein and the cholesterol pathway in AMD development [480]. Nevertheless, the Age-Related Eye Disease Study 2 (AREDS2) trial (NCT00345176) was unable to demonstrate protective effects for lutein or zeaxanthin in patients with advanced AMD [481]. Lutein and zeaxanthin improve retinal antioxidant capacity by modulating the expression of G-protein-coupled receptors and growth factors in rats [225] by diminishing inflammation in models of uveitis, choroidal neovascularization, diabetes, or ganglion cell damage, providing photoreceptor protection and reducing ER stress in rodent models of RP [224]. 

#### 3.1.9. Catechins 

Catechins are polyphenolic antioxidants that are found in many plants. (−)-Epigallocatechin gallate (EGCG) is the most abundant catechin-based flavonoid in green tea (*Camellia sinensis* L.). Its multiple actions include antioxidant, anti-inflammatory, neuro- and cardioprotective, antimicrobial and anti-carcinogenic actions, and it thus has therapeutic potential against different human diseases [482]. EGCG has an important scavenging activity and protects against H_2_O_2-_-triggered oxidative damage [483]. EGCG is hydrophilic, has a low molecular weight, and can cross the blood–retina barrier to reach the retinal cells and the entire ocular structure [484]. Overall, these properties make EGCG a potential treatment for retinal neurodegeneration. EGCG administration induced *Sod1* and *Gpx3* expression in the rat retina but suppressed *Cat* expression [484]. EGCG has shown retinal protection in models of retinal damage, including RP models, by reducing the levels of peroxidized lipids and nitrosative damage, and increasing total antioxidant capacity. These effects might be related to different mechanisms, including an antagonist’s effect on the NMDA receptor as a glutamate receptor antagonist, and reducing glutamate excitotoxicity by increasing Ca^2+^ influx through a signaling cascade involving protein kinase C [485,486,487]. EGCG modulates the gene expression of pro-oxidant and antioxidant enzymes and inhibits the expression of p38-MAPK and NF-κB. Its anti-inflammatory activity is related to the regulation of pro-inflammatory and anti-inflammatory factors, including interleukins, chemokines, TNF-α, NF-κB and COX-2 [482,488].

#### 3.1.10. Wolfberry-Derived Zeaxanthine Dipalmitate 

Wolfberry or *Lycium barbarum* (commonly known as goji berries) has been used for thousands of years in traditional Chinese medicine and is distinguished by its high antioxidant potential. The bioactive compounds of this exotic “berry” (polysaccharides, carotenoids or flavonoids, and phenolic compounds, among others) have been shown to modulate antioxidant, anti-inflammatory and antiapoptotic pathways, and to exhibit neuroprotective effects in retinal diseases in animal models and human subjects [489]. The carotenoid zeaxanthin dipalmitate [(3R,3′R)-3,3′-O-dipalmitoyl-β-carotene] is a major constituent in wolfberry and is considered as a promising supplement to delay RP, as evidenced by its beneficial morphological and functional effects in the retina of rd10 mice [490]. The mechanisms of action of zeaxanthin dipalmitate include the modulation of numerous genes in the STAT3, CCL2 and MAPK pathways, ultimately ameliorating the developing inflammatory processes (for a review, see [431]).

*Lycium barbarum* L. has recently been administered to patients with RP in a 12-month intervention CT (NCT02244996) [317], and was found to preserve the macular layer. 

#### 3.1.11. (Z)-7,4′-Dimethoxy-6-hydroxy-aurone-4-O-b-glucopyranoside

(Z)-7,4′-Dimethoxy-6-hydroxy-aurone-4-O-b-glucopyranoside (DHAG) is a compound isolated from the endophytic fungus *Penicillium citrinum* of mangrove plants with potent neuroprotective activity against oxidative damage [491]. The administration of DHAG in the rd10 mouse model by daily gavage from postnatal day 12 (P12) to P33 (rod cell death begins around P18 and is almost complete by P45) significantly improved photoreceptor survival [322]. DHAG reduced photoreceptor cell apoptosis in the rd10 retina by increasing the abundance of the antiapoptotic protein Bcl-2 and reducing the pro-apoptotic protein Bax and caspase 3/9 activities. In addition, DHAG activated Nrf2, which further triggered the expression of antioxidant genes to restore oxidative homeostasis, evidenced by the decreased protein expression of NOX1, reduced levels of MDA and ROS, and increased SOD activity and GSH levels. Furthermore, DHAG treatment inhibited inflammatory responses in rd10 mouse retinas, characterized by the decreased gene expression of *Il1b*, *Il6* and *Tnfa*, and pro-inflammatory expression of NF-κB and p38-MAPK.

#### 3.1.12. NRF2 

NRF2 is the master transcriptional regulator of antioxidative responses in multiple tissues, including the retina. Adeno-associated virus (AAV) has emerged as the vector of choice for gene delivery to the retina, and several laboratories have tested the therapeutic potential of AAV-mediated gene overexpression to protect against oxidative stress. AAV-mediated *Nrf2* gene delivery in the neural retina and RPE promoted the survival of cones and RPE cells and retention of vision in various mouse models of RP [492] and acute nerve damage [493]. Similarly, the subretinal injection of human oxidation stress resistance-1 (AAV8-hOXR1) in rd1 mice significantly improved photoreceptor survival and light response, delaying retinal degeneration [494]. *OXR1* is a key player in protecting against oxidative stress and exerts its neuroprotective functions by directly upregulating the expression of myriad antioxidant genes, or by controlling the transcription factors that regulate genes involved in oxidative stress-resistance [495].

#### 3.1.13. Multi-Target Iron Chelators 

Combination therapies that target complementary mechanisms to promote synergism have shown promise for retinal diseases. Multi-target iron-chelating compounds, including VK28, M30 and VAR10303, have been shown to exert neuroprotective effects in the rd10 mouse retina via synergistic antioxidant, anti-inflammatory and antiapoptotic mechanisms, which morphologically and functionally preserve photoreceptors, consequently, maintaining the visual function in RP retinas [323,324].

#### 3.1.14. Mitochondrial Nutrients and Metabolic Intermediates 

Photoreceptor cells are highly metabolically active and require sufficient supplies of ATP, NADPH, and metabolites to ensure proper functioning. Accordingly, mitochondrial dysfunction and metabolic dysregulation play critical roles in the pathogenesis of retinal degenerative disorders [496,497]. Many genes associated with IRDs directly or indirectly affect the metabolic pathways and mitochondrial function. Importantly, the death of rod photoreceptors, which constitute approximately 70% of all cells in the retina of most mammals, causes an increase in the release of RONS in the outer retina, resulting in damage to cones [498]. Many efforts have been made to prolong cone survival and improve visual acuity after prominent rod loss. Recently, an α-arrestin family member protein encoded by *Txnip* was shown to be beneficial in RP retinas (AAV-delivery of *Txnip*) by rescuing cone cells through enhancing their lactate catabolism and mitochondrial health [499]. Along the same line, mitochondrial cofactors, also known as mitochondrial nutrients, have been utilized to alleviate retinal disease and protect retinal cells against the pro-oxidant and dysmetabolic state. Examples of these are alpha-lipoic acid, coenzyme Q10 and carnitine [500]. Numerous studies have shown that exogenous creatine is very effective in protecting cells from oxidative stress damage [501], and dietary creatine supplementation augments cone survival in rd1 retinas and improves visual function [327]. It is now recognized that many interdependent cellular metabolism and energy pathways are defective in IRDs. Thus, new treatments aiming to strengthen or restore the impaired mitochondrial function or to replenish metabolic insufficiencies by dietary supplementation are receiving increasing interest [502,503].

### 3.2. Antiapoptotic Agents

#### 3.2.1. Synthetic Bile Acids: Ursodeoxycholic Acid and Tauroursodeoxycholic Acid

Ursodeoxycholic acid (UDCA) and its taurine conjugate derivative tauroursodeoxycholic acid (TUDCA) are bile acids with proven neuroprotective [365,504] and antiapoptotic, anti-inflammatory and antioxidant [505] actions. TUDCA exerts antiapoptotic effects in different models of retinal degeneration, not only photoreceptor loss secondary to IRD, oxidative stress or retinal detachment [361,363,364,365,366,367,504,506,507,508] but also in glaucoma [369] and other ocular diseases [509]. Its antiapoptotic actions are thought to involve the suppression of caspase-dependent and -independent pathways and reductions in ER stress. The main limitations of TUDCA as a potential therapy are the high doses that are needed and the limited bioavailability to the retina, which can be solved using different drug-delivery systems [507] with good results in animal models [510]. 

UDCA and TUDCA have been used in several models of neurodegenerative disease, and were found to preserve anatomy and function. Three CTs have evaluated their safety and efficacy to date, albeit for amyotrophic lateral sclerosis [511,512,513], with all showing that the drugs were well-tolerated and may have utility in slowing disease progression. To date, there is only one phase-1 CT with UDCA registered for the treatment of rhegmatogenous retinal detachment (NCT02841306).

#### 3.2.2. Progesterone

Progesterone and other steroid hormones such as estrogen have demonstrated neuroprotective effects in animal models of IRD [514]. Progesterone increases the expression of antiapoptotic proteins (Bcl-2, Bcl-xL) and reduces the expression of pro-apoptotic factors (Bax, Bad, caspase-3) [515]. It also exhibits anti-inflammatory actions by decreasing microglial and macrophage activation [516,517] and reduces the levels of inflammatory cytokines [518]. It can also diminish excitotoxicity [519,520].

Progesterone has been shown to preserve photoreceptor degeneration in animal models of RP and light-induced damage [380,381,383]. However, its translation to human studies is challenging, especially regarding doses, which might limit the development of CTs for IRDs. Nevertheless, progesterone has been tested in a phase-3 CT for traumatic brain injury (NCT00822900, Progesterone for the Treatment of Traumatic Brain Injury III [ProTECT]), but with no evident benefits over placebo.

### 3.3. Other Compounds (Nutraceuticals and Compounds with Mixed Mechanisms of Action)

#### 3.3.1. Vitamin A and E

Vitamins A and E have vital functions in photoreceptor maintenance. Given the evidence that patients with RP who take vitamin A and E and other nutrients show an attenuated decrease in ERG responses, several CTs have been launched to test the effectiveness of vitamin supplementation. One CT was developed using high doses of vitamin A to test for improvements in cone function measured with ERG (NCT00065455). The study was completed in 2009, but the results have not been published. Another CT investigated supplementation with both vitamins A and E (NCT00000114) [425]. These results were published in 1993. The authors treated 601 patients and found a beneficial effect of 15,000 IU/d of vitamin A on the disease course, but an adverse effect of vitamin E (400 IU/d) [439]. The same authors conducted a second CT in patients receiving vitamin A (15,000 IU/d) supplemented with DHA (1200 mg/d), which did not slow the disease course over a 4-year period [440].

Finally, the same authors tested the addition of lutein (12 mg/d) to patients with RP receiving vitamin A (NCT00346333), and reported that lutein slowed the loss of midperipheral visual field in non-smoking patients [430].

#### 3.3.2. Docosahexaenoic Acid 

DHA is the major polyunsaturated fatty acid in the retina, accounting for 50–70% of the fatty acids of the photoreceptor outer segment. DHA has an important role in the maintenance of retinal structure and function [521], not only in neurons but also in retinal vessels, and is necessary for phototransduction. DHA is also related to the increased production of GSH [430]. 

DHA metabolism is believed to be altered in retinopathies, due to pathogenic mutations in *ABCA4*, although no positive results have been found in patients treated with DHA supplements. For example, MacDonald and Sieving published the results of DHA supplementation in 11 patients with Stargardt disease, reporting no changes in macular function [441].

Several CTs have evaluated the effect of DHA supplementation in X-linked RP (XLRP). Hoffman and colleagues tested the ability of supplemented DHA (400 mg/d) to increase blood DHA levels and slow disease progression in 23 patients with XLRP. Results showed elevated DHA blood levels in patients receiving DHA, but no change in cone function measured by ERG versus placebo, although some benefits in rod/cone function were observed in subset analysis [522]. The same authors developed a phase-2, 4-year, follow-up CT (NCT00100230) including 78 patients receiving placebo or DHA. In this study, DHA was unable to slow the loss of ERG function in XLRP [436], although an analysis of ancillary visual function outcomes revealed a reduced rate of progression in dark-adapted thresholds and visual field sensitivities [438]. The authors suggested improvements in the study design, with a higher sample size and longer trial period, targeting a higher DHA blood level. Along this line, the authors found minor gastrointestinal discomfort in patients receiving 30 mg/kg DHA, and plasma and red blood cell DHA levels increased 4.4- and 3.6-fold, respectively, at year 4 [437]. A similar CT (NCT00004827) was designed to evaluate the potential of DHA supplementation to normalize DHA levels in red blood cells and retard the progression of visual function loss in patients with XLRP. The trial is completed but the results have not yet been published.

In a sub-group analysis of their original study, Berson et al. followed 221 patients receiving either vitamin A or vitamin A + DHA, finding no changes in the course of degeneration [440]. However, in those patients taking vitamin A prior to entry in the trial, the addition of DHA slowed the course of the disease for the first 2 years [440], but these differences were not preserved in years 3 and 4. 

Macular degeneration omega-3 study (MADEOS) is a CT in the recruiting phase, testing the efficacy of omega-3 fatty acids in AMD and *ABCA4*-associated retinopathy. 

Schwartz et al. performed a systematic review to evaluate the effect of vitamin A and fish oils in preventing the progression of RP [422]. They concluded that there is no clear evidence for treatment with either DHA alone or associated with vitamin A in the studied randomized CTs.

### 3.4. Drugs Interacting with Vitamin A in ABCA4-Associated Retinopathy

*ABCA4*-associated retinopathy is a highly prevalent IRDs. While no approved therapy is available to date, several therapeutic approaches have been investigated, including cell- and gene-based strategies. Different compounds with a variety of actions have been tested for therapeutic intervention in *ABCA4*-associated retinopathy [523]; some are related to antioxidant and anti-inflammatory actions, and others are based on the ability to halt/slow lipofuscin accumulation. Most studies use high doses of oral administration to reach the retina, which may lead to secondary effects. Here, we include not only antioxidants and inflammatory drugs, but also visual cycle modulators. 

A common target for these approaches is N-retinylidene-N-retinylethanolamine (A2E), a major component of lipofuscin and a by-product of the retinoid visual cycle. The alcohol–dehydrogenase-inhibitor 4-methylpyrazole (4-MP) can inhibit the visual cycle in rodents and has been used in animal models to delay dark adaptation. 4-MP has been tested in a CT (NCT00346853) but failed to delay dark adaptation [442]; however, it did prevent the accumulation of lipofuscin by delaying the processing of vitamin A derivatives. 

Another drug tested to prevent lipofuscin formation is ALK-001 (C20-D3-retinyl acetate), which is a deuterated form of vitamin A, slowing down vitamin A dimerization and A2E production. In mice, it diminishes the formation of A2E dimers and slows lipofuscinogenesis [524]. Based on these results, a phase-1 CT was launched in 40 healthy subjects (NCT02230228). There is an ongoing extension phase-2 CT examining the tolerability and effects of ALK-001 on Stargardt disease (TEASE), with 140 patients affected by *ABCA4*-associated retinopathy (NCT04239625), and another CT is in the recruiting phase (NCT02402660). The same drug is being used in a phase-3 study of geographic atrophy (NCT03845582).

#### 3.4.1. Emixustat 

The accumulation of toxic retinal by-products generated in the visual cycle by RPE65 can drive blindness. The generation of these products, including A2E, can thus be modulated by blocking RPE65 activity, which is a rate-limiting step in the visual cycle. Two such RPE65 inhibitors are emixustat and MB001. Emixustat hydrochloride (also known as ACU-4429) is a non-retinoid drug that exerts an inhibitory effect on RPE65, thus reducing the accumulation of vitamin A-based toxins. The inhibition should, however, be partial, to permit chromophore regeneration [525]. 

Emixustat and fenretinide, another visual cycle modulator, have been tested for the treatment of non-exudative AMD by oral administration [526]. In terms of safety, in a phase-1, placebo-controlled study (NCT00942240), emixustat had minimal systemic adverse events in healthy individuals, with 67% showing mild ocular side effects, which disappeared after withdrawal [527]. In patients with AMD, emixustat had a dose-dependent, reversible effect on rod function with dose-related mild and moderate ocular adverse events (NCT01002950) [528]. In a phase 2b/3 CT (NCT01802866, SEATTLE), emixustat failed to reduce the growth of the geographic atrophy [529]. Emixustat is currently being evaluated as a potential treatment in *ABCA4*-related retinopathy. The CT NCT03033108 (Pharmacodynamic Study of Emixustat Hydrochloride in Subjects with Macular Atrophy Secondary to Stargardt Disease) is examining 23 patients with Stargardt disease, treated with emixustat and assessed for ERG changes and adverse effects. Likewise, the CT NCT03772665 (SeaSTAR) is a phase-3 study including 194 patients (for 24 months), with an estimated study completion date of June 2022.

#### 3.4.2. Zimura 

The complement cascade is key to the immune response. Zimura (also known as avacincaptad pegol) is an aptamer that can inhibit the activity of complement factor C5 [530]. The proteolysis of C5 generates two fragments: C5a, an anaphylotoxin, and C5b, needed for the assembly of the membrane attack complex (MAC). The MAC can form a channel that penetrates the cell membrane of pathogens or damaged host cells, leading to cell death. Both C5a and MAC are implicated in tissue damage secondary to inflammation [531,532]. The inhibition of proteolytic C5 activation is a potential therapeutic approach in diseases that involve the overactivation or dysregulation of the complement pathway, such as certain retinal degenerative diseases [533]. 

Intravitreal administration of Zimura is being studied in both non-exudative AMD (NCT04435366) and in a phase-2 CT associated with ranibizumab in exudative AMD. NCT03364153 is a phase-2 CT used to evaluate the safety and efficacy of Zimura in 120 patients with Stargardt disease. 

#### 3.4.3. Soraprazan 

Soraprazan is a fast-acting inhibitor of H^+^,K^+^-ATPase and is used in gastroesophageal reflux disease [534]. It is a small molecule that can remove lipofuscin from RPE cells. It has been tested in an animal model of lipofuscinogenis [535] and a multi-national, multi-center, double-masked, placebo-controlled, proof-of-concept trial is evaluating the safety and efficacy of oral soraprazam in Stargardt disease (EudraCT 2018-001496-20).

#### 3.4.4. STG-001 

STG-001 is an indirect visual cycle modulator that reduces plasma concentrations of retinol-binding protein 4 (RBP4), the major retinol transporter, and slows down the visual cycle and the accumulation of cytotoxic retinoids in the retina. RBP4 is correlated not only with retinal disease but also with cardiovascular diseases, insulin resistance related to type 2 diabetes mellitus, and obesity. Two RBP4 antagonists are currently in CTs. One of them, STG-001, is being tested in patients with Stargardt disease in a phase-2a CT investigating two different doses of STG-001 (NCT04489511). 

## 4. Conclusions

Most IRDs, irrespective of the cause of the disease, result in photoreceptor loss and vision deterioration. Oxidative stress is a major contributor to cell death in photoreceptor cells, as they are constantly exposed to light and have a high metabolic demand to maintain normal function. Importantly, photoreceptors are post-mitotic cells and are unable to repair molecular damage through cell division, making these cells particularly vulnerable to molecular damage, metabolic alterations, genetic modifications, and environmental factors. Cell loss in IRDs leads to inflammatory responses that exacerbate the degenerative processes of the retina. A multitude of treatments aim to prevent, halt, or slow down the neuronal degeneration underlying IRDs by boosting the endogenous antioxidant defense system and/or providing exogenous antioxidant agents. Similarly, a number of anti-inflammatory therapies are being investigated.

Maintaining cell health is imperative to allow for the activation of intrinsic cytoprotective mechanisms to combat stress or damage and prevent disease progression. The healthier the status of retinal cells, the better the response to therapeutical approaches. Numerous therapeutic interventions are being tested at present to maintain cellular homeostasis by enhancing oxidative defenses and mitochondrial function to prevent ROS-induced damage linked to dysregulated signaling pathways. These strategies focus on targeting ROS production, aiming to decrease its levels and promote cell protection. However, several challenges need to be overcome to successfully achieve this goal. A key point to consider when treating IRDs is that there is an unstable balance between the antioxidant effect of the administered compounds and the total antioxidant capacity of the patients. Moreover, the additive effect of different drugs with different mechanisms of action does not always result in enhanced cell survival, possibly due to the interactions between different cellular pathways. Finally, the causative mutation of the disease can promote different responses to the same drugs in patients. 

New or improved modalities of gene- and cell-based therapies are being developed to treat IRDs. Gene therapy has proven efficacy and is already in clinical use. The functional recovery of cells that have been genetically modified is very encouraging for patients; however, inflammation and microglia activation through oxidative stress persists in untreated areas, potentially compromising the long-term success of the therapy. A degenerated retina with a complete loss of vision needs to be healthy to maintain non-visual functions such as the control of circadian rhythms and pupil contraction. Thus, the maintenance of retinal cell health using neuroprotective compounds, in combination with gene therapy, may be key to long-term therapy success. The ability to maintain inflammation within normal ranges and finding the correct balance between endogenous and exogenous antioxidants in patients with IRDs are exciting challenges.

A successful therapeutical approach to IRDs will require a profound knowledge of the etiopathogenesis of the disease, the causative mutation, and the efficacy of drug treatments. Potential drug compounds should display good safety and tolerability profiles and be capable of crossing the blood–retinal barrier (providing suitable bioavailability in the retina) without the need for high systemic doses or, alternatively, be suitable for intraocular use.

## Figures and Tables

**Table 1:** Antioxidant, antiapoptotic and anti-inflammatory compounds with demonstrated efficacy in retinal disease. The table compiles proven therapeutic interventions for inherited and induced retinal damage models.

Compounds	Animal Models of Photoreceptor Degeneration, Diabetic Retinopathy or Retinal Ganglion Cell Death	Clinical Use in Ophthalmology
Antioxidant and anti-inflammatory		
**N-acetylcysteine**	IRD and retinal damage models: Rd10 mouse [108,109] P23H rats (studying NLRP3 inflammasome) [59] Blue-light damage in mice [110] Light damage in mice (with angiotensin II type 1 receptor blockade) [111] Light damage in zebrafish [112] Cypermethrin-induced damage in rats [113] AMD: Choroidal neovascularization in mice (studying plasma-activated medium) [114] Autoimmune uveitis in mice [115,116] Diabetic mice (Redd1−/−) [117] Diabetic rats [118] Mouse normal tension glaucoma [119] Choroidal neovascularization in rats [120]	IRD: RP: NCT03063021 (FIGHT-RP1), NCT04864496 (NAC) [121,122] AMD: NCT03919019 (Macuprev) [123]
**N-acetylcysteine** **amide**	IRD and retinal damage models: Rd10 mice [124] Light injury in mice [125]	IRD: Usher syndrome: NCT04355689 (SLO RP)
**Thioredoxin**	IRD and retinal damage models: Rd1 mice [126] Nxlnl1−/− mice after light damage [127] DR models: Diabetic mice with light damage [128] Ganglion cell damage models: Perinatal hypoxia-ischemia retina damage in rats	
**Saffron**	IRD and retinal damage models: Light-induced photoreceptor degeneration in rats [129,130,131] P23H rats [132] NMDA-induced damage in mice [133]	IRD: Stargardt disease: NCT01278277 (STARSAF02) AMD: NCT00951288 [134]
**Minocycline**	IRD and retinal damage models: Rhodopsin−/− mice [135] (Mertk)−/− Cx3cr1GFP/+ Ccr2RFP/+ mice [136] P23H and RC rats [137] rd10 mice [138] Rds mice [139,140] NMDA-induced damage in mice [141,142] S100B retina degeneration model [143] Branch retinal vein occlusion in rats [144] Lipofuscinosis: Cln3Δex7/8 mouse [145] Focal light damage in mouse [146] Light damage in mice [147,148] Light damage in rats [149] AMD models: Aging: Chx10-Cre;Tsc1fx/fx mouse (Tsc1-cKO) [150] Subretinal hemorrhage in mouse [151] DR models: Diabetes rats (histone levels) [152] Diabetes rats (STZ) [153,154] Ganglion cell damage models: Ischemia/reperfusion mice [155,156] Ischemia/reperfusion rats [157,158,159] Glaucoma model in rats [160,161] and mice [162] DBA/2J mouse model of glaucoma [163,164] Optic nerve transection in rats [156] and mice [155] Optic nerve crush mice [165] Axotomy in rat [166]	IRD: RP: NCT04068207; NCT02140164 AMD: NCT02564978 NCT00893724 DR: Diabetic macular edema: NCT01120899
**Melatonin**	IRD and retinal damage models: P23H rats [167,168] Rd10 mice [169] Rds mice [170] Light damage in mice [171] MNU-induced photoreceptor degeneration in mice [172] Toxoplasma retinochoroiditis in SD rats (melatonin + zinc) [173] AMD models: Laser-induced CNV in mice [174] Non-exudative AMD after cervical ganglionectomy in mice [175] DR models: STZ rats [176,177,178,179,180,181,182,183] STZ-nicotinamide rats [184] High-fat diet + STZ in mice [185] For review [186] Ganglion cell-damage models: Hypoxia-ischemia mice [187] Ischemia-reperfusion guinea pig [188]	AMD: Effect of melatonin on AMD [189] Macular damage with blue filtering IOL: NCT00444249 DR: NCT04547439 NCT03478306
**Curcumin**	IRD and retinal damage models: P23H swine model of RP [190] P23H rat [191] MNU-induced photoreceptor apoptosis in SD rats [192] Light-induced retinal degeneration in rats [193] DR models: Diabetic rats [194,195,196,197,198,199,200,201,202,203,204] Rabbit model of proliferative retinopathy [205] Other retinal diseases models: CLN6 (neuronal ceroid lipofuscinosis) mice [206] Ganglion cell damage models: Rat ischemia/reperfusion [207,208] Retinal ischemia/reperfusion in a rat stroke model [209] Chronic methanol intoxication in rats [210]	AMD: NCT04590196 NCT05062486 (resveratrol + quercetin + curcumin) DR: Chronic diabetic macular edema [211] In DR, NCT04378972 (curcumin + homotaurine + vitamin D3) In diabetic macular edema, NCT03598205 (Curcumin + dexamethasone)
**Quercetin**	IRD and retinal damage models: Rd10 mice (+ naringenin) [212] P23H rats Light damage in mice (quercertin + myricetin) [213] Blue-light damage in Balb-c mice [214] Light damage in rats [215] AMD models: Nrf2−/− mice [216] DR models: Zebrafish model of DR [217] Diabetic rat retina [218,219] Other retinal disease models: Rodent model of retinopathy of prematurity [220] Ganglion cell damage models: Chronic glaucoma rat model [221] Ischemia/reperfusion in rats [222]	
**Lutein (L)** **Zeaxanthin (Z)**	IRD and retinal damage models: Light damage retinopathy in quails (L) [223] and rodents (Z&L) [224,225] Rd1 mice (L + Z + lipoic acid + glutathione + Lycium barbarum) [226] Pde6b rd10 mice [224] Light-induced retinopathy (L&Z) [227] DR models: Diabetic rats, zeaxanthin [228] Light-exposed retinas of mice [229] Other retinal disease models: Inflammatory state of retina in obesity-induced high-fat diet [230] Sod2−/− mice (Z) [231] Vldlr−/− mice [232] House finch vision with carotenoids supplementation (Z or astaxanthin) [233]	AMD: NCT03919019; NCT00121589; NCT00527553; NCT00564902; NCT01269697; NCT01648660; NCT00763659; NCT01646047; NCT04741763; NCT01694680; NCT00879671 (L) Dietary supplement for AMD: NCT04496817; NCT00345176; NCT01404845; NCT00902408 (L); NCT01527435 (Z); NCT02287298 (Z); NCT02113254 (Z) Aging: NCT02147171 DR: NCT04496817; NCT01627977 Multiple compounds: NCT04117022; NCT04071977; NCT03702374 Other diseases: Corioretinopathy: NCT00963131 Albinism: NCT02200263 Juxtafoveal telangiectasia: NCT01354093 Glaucoma: NCT04460365; NCT03959592; NCT01646047 (Multiple compounds)
**Catechins**	IRD and retinal damage models: P23H1 rats [234,235] Light-induced photoreceptor degeneration in mice [236] Light damage in albino rats [237] Oxidative damage by SNP injection in rats [238] Sodium-iodate-induced retinal degeneration in rats [239] Photoreceptor apoptosis by injection of MNU in SD rats [240] NMDA excitotoxicity in rats [241] DR models: Diabetic rats [242] Ganglion cell damage models: Ischemia/reperfusion in rats [243] Glaucoma model in mice [244] Ischemia/reperfusion in albino rabbits [238]	AMD: NCT03205202 (Cocoa supplement with 80 mg epicatechins) Supplementation with flavonoids (epigallocatechin, quercetin, etc.) [245]
**Resveratrol**	IRD and retinal damage models: Rd10 mice [246] Light-damaged rat retinas [247] Light damage, mouse [248] Zebrafish model of retinal neurodegeneration by NMDA [249,250] AMD and CNV models: Aging zebrafish retinas [249] Aged SAMP8 mice [251] Choroidal neovascularization mouse model [252]; resveratrol + omega 3 [253] DR models: STZ-induced diabetes in mice [254,255,256] Diabetic rats [257,258,259,260,261,262,263,264] Other retinal disease models: Retinal detachment in Brown Norway rats by subretinal injection of sodium hyaluronate [265] Induced myopia in golden Syrian hamsters [266] Vldlr−/− mice, model of macular telangiectasia [267] Rats with oxygen-induced retinopathy of prematurity [268] Oxygen-induced retinopathy model, SD rats [269] Ganglion cell-damage models: Mouse model ischemia/reperfusion [268,270,271,272] Ischemia/reperfusion Sprague-Dawley rats [273,274,275,276] Mouse model ocular of hypertension (ischemia/reperfusion) [277] Rat chronic ocular hypertension model [278] Steroid-induced ocular hypertensive rats [279] Glaucoma model by injecting hyaluronic acid, Wistar albino rats [280]: riluzole + resveratrol Optic nerve crush in mice [281] Optic nerve transection in SD rats [282] Uveitis models: Inflammation model by LPS injection in mice [283]	NCT02321176 (pharmacokinetics of resveratrol in the eye) AMD: NCT02625376; NCT04756310: supplementation in AMD [284] Observer-blinded trial in wet AMD patients (lutein, zeaxanthin, resveratrol, hydroxytyrosol and DHA+ the AREDS EU recommended doses) [284] NCT05062486: resveratrol + quercetin + curcumin for AMD Case report (resveratrol + lutein + Vac. myrtilus) in AMD patient [285] Octogenarians + resveratrol supplement [286] DR: Observational study with diabetic patients. Supplementation with multinutrient complex (resveratrol + vitamins, L, Z, etc.) [287] NCT04117022: diabetic retinopathy (rich formula) NCT03866005: Adjunctive Carotenoids Plus Antioxidants in Anti-VEGF Treated Diabetic Macular Edema (PROACTIVEDME)Other:Effect on choroidal thickness, in young and healthy:NCT02321189
**Dietary antioxidants**	IRD and retinal damage models: Tested individual effects of vitamins in dystrophic RCS rats [288] LED light damage rats (β-Cryptoxanthin) [289] Light damage in SD rats (AREDS ± antioxidants) [290] Light damage in rats (suppl antioxidants and omega 3) [291] Light damage in rats (Beta carotene) [292] Light rabbit (blueberry polyphenols) [293] AMD and CNV models: CNV mice (Resvega: omega 3 + resveratrol) [252] 𝛽Amyloid mice (grape seed extracts) [294] β5−/− mice [295] Other retinal disease models: Smith–Lemli–Opitz syndrome (defective cholesterol synthesis) [296] ApoE−/− mice. Suppl L vs. multivitamin [297] DR models: Diabetes, rats (ascorbic acid, vitamin E, beta-carotene, zinc, and copper) [298] Diabetes rats (carrot powder) [299] Diabetes and experimental galactosemia in rats (antioxidants, ascorbic acid, alpha-tocopherol) [300] Diabetes and experimental galactosemia in rats VI (ascorbic acid + trolox + alpha-tocopherol + NAC+ beta-carotene + selenium) [301] Ganglion cell damage models: Ischemia/reperfusion mice with ubiquinol [302] Pressure trauma in mice [303]	AMD: AMD prevention [304] NCT03326401; NCT04756310; NCT02264938; NCT00121589; NCT00800995 NCT00000145 (AREDS); NCT00345176 (AREDS2) DR: NCT04496817 Other diseases: Retinopathy of prematurity NCT03866005 Hyperoxia-induced retinal reduced blow flow: NCT00712907 Oxygen-induced retinal vasoconstriction: NCT02221089ithout DR
**Fructus lycii**	IRD and retinal damage models: Rd10 mouse [305] Methyl-N-nitrosurea photoreceptor degeneration mouse model Rd1 mice (L + Z+ lipoic acid + glutathione + Lycium barbarum) [226] Light damage in rats [306] Light damage in mice [307] Intravitreal paraquat in rats [308] DR models: Diabetic rats [309] Other retinal diseases models: Alzheimer’s Disease Model Mouse Retina [310] Ganglion cell damage models: Ischemia/reperfusion in rats [311] Acute hypertension in mice [312,313] Ocular hypertension in rats [314,315,316]	IRD: RP: NCT02244996 (Lycium barbarum) [317] AMD: Goji berry intakes and macular pigment in healthy adults (randomized pilot study) [318]
**Alpha-lipoic acid**	IRD and retinal damage models: Rd1 mice (L + Z + lipoic acid + glutathione + Lycium barbarum) [226] Rd1 mouse (mixture) [319]. Rd10 model (α-tocopherol, ascorbid acid, α-lipoic acid) [320] Q334 model (α-tocopherol, ascorbid acid, α-lipoic acid) [320] Rd1 model (LA and/or progesterone) [321]	AMD: NCT02613572 DR: NCT01880372
**(Z)-7,4′-Dimethoxy-6-hydroxy-aurone-4-O-** **β** **-glucopyranoside (DHAG)**	IRD and retinal damage models: Rd10 mouse [322]	
**Multi-target iron chelators**	IRD and retinal damage models: Mouse model of RP [323] Rd 10 mice [324,325] NMDA damage in rats [326]	
**Creatine**	IRD and retinal damage models: Rd1 mice [327]	Gyrate atrophy with hyperornithinaemia (4 patients) [328] Gyrate atrophy (7 patients) [329]
**Sulforaphane**	IRD and retinal damage models: Phd6b rd10 mice [330] Light damage mice (sulforaphane induces TRX) [331] Light damage mice [332] Tubby mouse [333] DR models: Diabetic STZ rats [334] Ganglion cell damage models: Rat model ischemia reperfusion [335] Ischemia in mice [336]	
**NOS inhibitors**	IRD and retinal damage models: Rd1 mice [337] S334ter-3 rat [338] Light damage in rats [339] Ganglion cell damage models: Rat model of chronic glaucoma [340,341] Rat axotomy [342]	
**Sigma1R ligand (+)-** **Pentazocine (PTZ)**	IRD and retinal damage models: Rd10 mice [343,344,345,346,347] NMDA damage in mice [348] DR models: Diabetic mice Ins2(Akita/+) [349,350] Ganglion cell damage models: Optic nerve crush in mice [351]	
**Norbixin** **(bixin extracted from Bixa Orellana)**	IRD and retinal damage models: Blue-light model of photodamage in rats [352] Abca4−/− Rdh8−/− mice [353]	
**(3R)-5,6,7-trihydroxy-3-isopropyl-3-methylisochroman-1-one**	IRD and retinal damage models: Pde6b rd10 mice [354]	
**Glycyrrhizic acid/Glycyrrhizin**	IRD and retinal damage models: IGFB-3 KO mouse [355] Blue-light-induced damage in mouse [356] DR models: Diabetic rats [357,358] Ganglion cell damage models: Acute model of glaucoma in mice [359] Ischemia-reperfusion in mice [360]	
**Antiapoptotic agents**		
**Tudca**	IRD and retinal damage models: P23H AD rat model [361] MNU-induced photoreceptor degeneration mouse model [362] Leber Congenital Amaurosis mouse [363] Rd1 mice [364] Rd10 mice [365] Rd1, Rd10, Rd16 (Bardet–Biedl Syndrome type 1) [366] Light-induced damage in mice [367] RPGR conditional knockout (cko) mouse [368] NMDA-induced damage in mice [369] AMD and CNV models: CNV laser-induced rat [370] DR models: Mouse model of type 1 diabetes [371] Ex vivo model of RD in rats [372] Other retinal diseases models: Retinal detachment rat model [373] Ganglion cell damage models: Rat optic nerve crush [374]	Others: Rhegmatogenous RD: NCT02841306 URSO
**Rasagiline**	IRD and retinal damage models: Rd10 mice (RP) [375] Prph2/rds mouse [376] Ganglion cell damage models: Glaucoma model in rats [377] Mouse, ischemia/reperfusion [378] (rasagiline + idebenone)	Others: Retinal detachment: NCT02068625 (Macula off- retinal detachment) [379]
**Norgestrel/** **Progesterone**	IRD and retinal damage models: Pde6b Rd10 mouse [380,381,382] Rd1 mice (L + Z + lipoic acid + glutathione + Lycium barbarum) Rd1 mouse [383] Rd10 mice [384] Acute light-induced degeneration model in mice [382,385] Ganglion cell damage models: Rat models of ocular ischemia [386] Review: [387]	
**Proinsulin**	IRD and retinal damage models: Rd10 mice [388] P23H RP rats [389]	Others: Glaucoma NCT05206877, NCT04118920
**Nutraceuticals and other compounds**		
**Anthocyanin** **Cyanidin-3-glucoside (C3G)** **Bilberry extract**	IRD and retinal damage models: Light-induced photoreceptor degeneration in rats (+L) [390] Light damage in rabbits [391] MNU-induced damage in rats [392] Other retinal disease models: Oxygen-induced retinopathy in mice [393] IRD and retinal damage models: Photo-stressed murine model [394] Light damage in rabbits [395] AMD models: OXYS rats [396] DR models: STZ rats [397] Other retinal disease models: Oxygen-induced retinopathy in mice [398] Ganglion cell damage models: Optic nerve crush in mice [399] Uveitis models: Endotoxin-induced uveitis in mice [400]	Metabolism and Clearance of Cyanidin 3 Glucoside: NCT01106729 NCT01942746: Blueberry Effects on Dark Vision and Glare Recovery [401]
**4-Phenylbutyric acid**	IRD and retinal damage models: Photo-stressed murine model [402] P23H mice [403] Leber congenital amaurosis mice [404] Other retinal disease models: Hypoxia mice [405] Ganglion cell damage models: Ischemic optic neuropathy in mice [406] Ischemic rats [407]	IRD: Achromatopsia NCT04041232
**Celastrol**	IRD and retinal damage models: Light-induced retinal degeneration mice [408] Ganglion cell damage models: Ocular hypertension in mice [409] Optic nerve crush [410]	
**Salvia miltiorrhiza Bunge**	IRD and retinal damage models: Light damage in mice [411] Rd10 mice (+ Fructus lycii) DR models: DR rats (FXST Chinese medicine + various compounds)	
**Vitamin A and/or E**	Vitamin A IRD and retinal damage models: Lrat−/−, Rpe65−/−, and Gnat1−/− mouse models, supplemented with Vit A derivatives [412] T17M mouse [413] DR models: DR rat [414,415] Other retinal pathology models: Obesity-associated retinal degeneration in WNIN/Ob rats [416] Vitamin E IRD and retinal damage models: Radiation induced retinal damage in a rat model [417] Light-induced damage in guinea pig [418] Light damage in albino mice [419] DR models: Diabetic rats (taurine, Vit E + selenium) [420] Other retinal pathology models: Porcine hypercholesterolemia [421]	Vitamin A IRD: RP: NCT00000116 (vitamin A + fish oils) [422]; NCT00346333 (L + vit A); NCT04499820 (flavonoids, L, Z) Choroidemia: NCT05045703 Stargardt disease (ALK-001): NCT02230228; NCT04239625; NCT02402660 LCA, RP Drug: QLT091001, NCT01014052 Night blindness in Sorsby’s fundus dystrophy [423] AMD: AMD Review [424] AMD, reticular pseudodrusen: NCT03478878 AMD: NCT03478865 Geographic atrophy: NCT03845582 AMD (Phase I): NCT02230228 Others: Retinopathy of Prematurity: NCT00417404; NCT03154723; NCT03779776 (Vit AD); NCT02102711 DR: NCT04000789 (Vit A, Vit E); NCT04000789 Vitamin E IRD: RP: NCT00000114 (Vit A + Vit E) [425] AMD: NCT00784225; NCT00000161; NCT00893724; NCT00893724; NCT00000145 (various antioxidants) DR: NPDR (various compounds) [426] Others: Glaucoma: NCT01544192 Retinopathy of Prematurity: NCT03274596

Abbreviations: IRD, inherited retinal dystrophies; AMD, age-related macular degeneration; DR, diabetic retinopathy; CNV, choroidal neovascularization; RP, retinitis pigmentosa; LCA, Leber Congenital Amaurosis; STZ, streptozotocin, MNU, N-methyl-N-nitrosourea; NMDA, N-methyl-D-aspartate; L, Lutein; Z, Zeaxanthin; NAC, N-acetylcysteine; DHA, docosahexaenoic acid; NOS, nitric oxide synthase.

**Table 2:** Clinical trials in IRD using molecules with antioxidant, antiapoptotic, anti-inflammatory and/or visual cycle modulator properties.

Compound	Mechanism	Disease	Participants (Number)	Follow-Up Time	Trial ID	Phase/ Status	References	Results
**N-acetylcysteine** **(NAC)**	Antioxidant Stabilizes protein structure	RP	30	10 m	NCT03063021 (FIGHT-RP)	1/ Completed (2019)	Campochiaro et al., 2020 and Kong et al., 2021 [121,122]	NAC is safe and well-tolerated Improvement in macular functioning cones
**NAC**		RP	30	6 m	NCT04864496	2/Active		
**NACA** **(NPI-001)**	Antioxidant Stabilizes protein structure	Usher syndrome	48	24 m	NCT04355689 (SLO RP)	1, 2/ Recruiting		
**Saffron**	Neuroprotective and antioxidant effects	Stargardt Disease	30 (31)	6 m	NCT01278277 (STARSAF02)	1,2/ Unknown	Piccardi et al., 2019 [427]	No detrimental effects on ERG and visual acuity
**Minocyclin**	Anti-inflammatory, antiapoptotic and neuroprotective effects	RP	35	24 wk	NCT04068207	2/ Recruiting		
**Minocyclin**		Cystoid macular edema associated with RP	7	12 m	NCT02140164	1, 2/ Completed (2015)	Cukras et al., 2017 [428]	Well tolerated. No significant changes in mean visual acuity. Small but progressive decrease in mean central macular thickness.
**Lutein**	Removes ROS; protection against photo-oxidative stress	RP	34	48 wk	NCT00029289	1,2/ Completed (2008)	Bahrami et al., 2006 [429]	Lutein supplementation improves visual field
**Lutein in patients receiving vitamin A**		RP	240	5 yr	NCT00346333	3/ Completed (2008)	Berson et al., 2010 [430]	12 mg/d of lutein slows visual field loss among nonsmoking patients with RP with vitamin A
**Lycium** **barbarum**	Antioxidative, anti-inflammatory, and antiapoptotic mechanisms	RP	50 (42)	1 yr	NCT02244996	NA/ Completed (2017)	Chan et al., 2019 and Vidović et al., 2022 [317] For review: [431]	Preservation of photopic vision
**4-Phenylbutyric acid**	ER stress-regulated transmembrane protein	Achromatopsia	2	6 m	NCT04041232	Early phase 1/ Not yet recruiting		
**Vitamin A**	Improvement in cone photoreceptor function	RP (RHO1 mutation)	10 (5 RP)	6 wk	NCT00065455	1/ Completed (2009)		
**Vitamin A and/or** **Vitamin E**		RP	601 (572)	4 yr.	NCT00000114	3/ Completed (1987)	Berson et al., 1993 [425]	Beneficial effect of 15,000 IU/d of vitamin A Adverse effect of 400 IU/d of vitamin E
**Vitamin A**		RP		5 yr	NCT00000116	3/ Completed (1997)		
**Vitamin A**		Choroideremia	10	8 m	NCT05045703 (DARC)	Not yet recruiting		
**QLT091001**	Replaces chromophore in visual cycle	Leber congenital amaurosis (mutation *RPE65*, *LRAT*)	32	12 m	NCT01014052 (RET IRD 01)	1b/ Completed (2012)	Scholl H et al., 2015 [432] Wen & Birch 2015 [433] Koenekoop et al., 2014 [434]	Improvements in visual field and/or visual acuity. Cortical activation
**QLT091001** **(retreatment)**		Leber congenital amaurosis (mutation *RPE65*, *LRAT*)	27	12 m	NCT01521793 (RET IRD 01 extension)	1/ Completed (2014)	Scholl et al., 2015 [435]	Sustained visual improvements
**QLT091001**		ADRP (*RPE65* mutation)	5	12 m	NCT01543906	1/ Completed (2014)		
**ALK-001**	Chemically modified vitamin A (replacement of vitamin A)	Healthy volunteers	40	4 wk	NCT02230228	1/ Completed		
**ALK-001**		Stargardt Disease	140	24 m	NCT02402660 (TEASE)	2/ Recruiting		
**ALK-001**		Stargardt Disease	140	24 m	NCT04239625 (TEASE-2, an open-label extension of TEASE)	2/ Enrolling by invitation		
**DHA (docosahexaenoic acid; omega 3)**	Key cell membrane component involved in multiple metabolic pathways	X-linked RP	221 (208)	4 yr	NCT00100230	2/ Completed (2014)	Hoffman et al., 2014 [436] Hughbanks- Wheaton et al., 2014 [437] Hoffman et al., 2015 [438]	
**DHA**		X-linked RP	46	3 yr	NCT00004827	2/ Completed (2002)	For review see: Schwartz et al., 2020 [422]	
**DHA**		Usher Syndrome	100		NCT00004345	NA/ Terminated		
**DHA in** **patients** **receiving** **Vit A**		RP	221	4 yr		NA	Berson et al., 2004 [439] Berson et al. 2004 [440]	No improvement with DHA in patients with RP receiving Vit A 4 yr Improvements in the first 2 yr
**DHA**		Stargardt or Stargardt-like Macular Dystrophy	22	15 m	NCT00060749	1/ Completed (2007)	MacDonald & Sieving 2018 [441]	
**DHA**		Stargardt and dry AMD	32	24 wk	NCT03297515 (MADEOS)	NA/ Completed (2020)		
**Hydroxychloroquine**	Targets autophagy pathway	P23H-RHO RP	12	18 m	NCT04120883	1, 2/ Recruiting		
**4-Methylpyrazole (4-MP) (alcohol dehydrogenase inhibitor)**	Slows down processing of vitamin A derivatives	Healthy volunteers	10	6 wk	NCT00346853	1/ Completed	Jurgensmeier et al., 2007 [442]	4-MP does not inhibit human visual cycle sufficiently to be evaluated for Stargardt disease treatment
**Emixustat**	RPE65 inhibitor	Stargardt	23	1 m	NCT03033108 (SeaSTAR)	2A Dose scalation/ Completed (2017)		No SAES Frequent ocular side effects
**Emixustat**		Stargardt	194	2 yr	NCT03772665	3/Active		
**Soraprazan**	H,K+-ATPase inhibitor Removes retinal lipofuscine accumulation	Stargardt	90	12 m	EudraCT 2018-001496-20	2/Active		
**Zimura (avacincaptad pegol)**	Aptamer that inhibits the activity of complement factor C5	Stargardt 1	120	18 m	NCT03364153	2b/ Recruiting		
**STG-001**	RBP4 antagonist Visual cycle regulator	Stargardt 1	10	4 wk	NCT04489511	2a (2 doses)/ Completed (2021)		
**L-DOPA**	Upregulates PDEF Downregulates VEGF	RP	50	5 yr	NCT02837640	2/NA		
**Valproic acid**	Neuroprotective Induces microglial apoptosis	ADRP	90	52 wk	NCT01233609	2/ Completed (2015)	Birch et al., 2018 [443]	No efficacy was found
**Valproic acid**		RP	200	48 wk	NCT01399515	2/ Completed (2015)		
**Dunaliella** **Bardawil powder (oral)**	Beta carotene	RP	34	1 yr	NCT01256697	NA/ Completed (2009)	Rotenstreich et al., 2013 [444]	Increase retinal function in RP
**Dunaliella Bardawil powder**		RP in adolescents	30	72 wk	NCT02018692	1, 2/Not yet recruiting		
**Dunaliella Bardawil powder**		RP	100	72 wk	NCT01680510	2, 3/ Recruiting		
**rhNGF** **(recombinant human nerve growth factor) (drops)**	Neuroprotection	RP	50	48 wk	NCT02110225	1, 2/ Completed (2015)		
**Cannabis (cannabidiol:THC, 1:1)**	Neuroprotection	RP	50	3 h.	NCT03078309	1/ Recruiting		
**NP-001 (oral)**	Inactivates macrophages. Anti-inflammatory	Usher syndrome	48	24 m.	NCT04355689 (SLO RP)	1, 2/ Recruiting		

Abbreviations: RP, retinosis pigmentaria; ADRP, autosomal dominant retinosis pigmentaria; h, hour(s); wk, week(s); m, month(s); yr, year(s). Clinical trial information was retrieved from ClinicalTrials.gov (http://www.clinicaltrials.gov, accessed on 1 February 2022) and EU Clinical Trials Register (https://www.clinicaltrialsregister.eu/, accessed on 1 February 2022).

## Data Availability

Not applicable.
